# 4-(4-Nitro­benzene­sulfonamido)pyridinium trifluoro­acetate

**DOI:** 10.1107/S1600536808021405

**Published:** 2008-07-16

**Authors:** Jiang-Sheng Li, Mei-Lian Fan, Wen-Sheng Li, Wei-Dong Liu

**Affiliations:** a School of Chemical & Biological Engineering, Changsha University of Science and Technology, Changsha 410076, People’s Republic of China; b College of Chemistry and Chemical Engineering, Hunan University, Changsha, Hunan 410082, People’s Republic of China; c Hunan Research Institute of Chemical Industry, Changsha 410007, People’s Republic of China

## Abstract

In the title compound, C_11_H_10_N_3_O_4_S^+^·C_2_F_3_O_2_^−^, the benzene ring makes an angle of 87.3 (2)° with the pyridinium ring. The nitro group is essentially coplanar with the benzene ring. The F atoms of the CF_3_ group are disordered over two positions with almost equal occupancy [0.531 (12)/0.469 (12)]. The crystal structure is stabilized by N—H⋯O and C—H⋯O hydrogen bonds.

## Related literature

For studies of supra­molecular chemistry involving pyridinium rings, see: Li *et al.* (2007 ▶). For 4-nitro-*N*-(4-pyrid­yl)benzene­sulfonamide, see: Yu & Li (2007 ▶). For its salt form, see: Zhou *et al.* (2008 ▶).
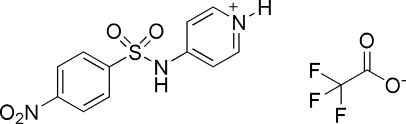


## Experimental

### 

#### Crystal data

C_11_H_10_N_3_O_4_S^+^·C_2_F_3_O_2_^−^
*M* *_r_* = 393.30
Monoclinic, 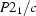

*a* = 5.662 (2) Å
*b* = 17.497 (7) Å
*c* = 16.173 (6) Å
β = 96.595 (7)°
*V* = 1591.5 (11) Å^3^
*Z* = 4
Mo *K*α radiation
μ = 0.28 mm^−1^
*T* = 294 (2) K
0.10 × 0.04 × 0.04 mm

#### Data collection

Bruker SMART 1K CCD area-detector diffractometer
Absorption correction: multi-scan (*SADABS*; Sheldrick, 1996 ▶) *T*_min_ = 0.973, *T*_max_ = 0.989
7696 measured reflections
2763 independent reflections
1596 reflections with *I* > 2σ(*I*)
*R*_int_ = 0.067

#### Refinement

*R*[*F* ^2^ > 2σ(*F* ^2^)] = 0.065
*wR*(*F* ^2^) = 0.150
*S* = 1.05
2763 reflections
269 parameters
43 restraints
H atoms treated by a mixture of independent and constrained refinement
Δρ_max_ = 0.40 e Å^−3^
Δρ_min_ = −0.33 e Å^−3^

### 

Data collection: *SMART* (Bruker, 1997 ▶); cell refinement: *SAINT* (Bruker, 1997 ▶); data reduction: *SAINT*; program(s) used to solve structure: *SHELXS97* (Sheldrick, 2008 ▶); program(s) used to refine structure: *SHELXL97* (Sheldrick, 2008 ▶); molecular graphics: *SHELXTL* (Sheldrick, 2008 ▶); software used to prepare material for publication: *SHELXTL*.

## Supplementary Material

Crystal structure: contains datablocks global, I. DOI: 10.1107/S1600536808021405/bt2740sup1.cif

Structure factors: contains datablocks I. DOI: 10.1107/S1600536808021405/bt2740Isup2.hkl

Additional supplementary materials:  crystallographic information; 3D view; checkCIF report

## Figures and Tables

**Table 1:** Hydrogen-bond geometry (Å, °)

*D*—H⋯*A*	*D*—H	H⋯*A*	*D*⋯*A*	*D*—H⋯*A*
N1—H1*A*⋯O6	0.899 (11)	1.812 (14)	2.706 (5)	173 (5)
N2—H2*A*⋯O6^i^	0.86 (5)	2.00 (5)	2.858 (5)	176 (5)
C2—H2⋯O5^i^	0.93	2.52	3.343 (6)	148
C3—H3⋯O1^ii^	0.93	2.56	3.406 (6)	151
C8—H8⋯O5^iii^	0.93	2.48	3.204 (6)	135
C10—H10⋯O3^iv^	0.93	2.39	3.184 (6)	143

Symmetry codes: (i) 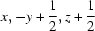; (ii) 
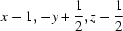
; (iii) 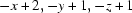; (iv) 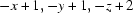.

**Fig. 1.**
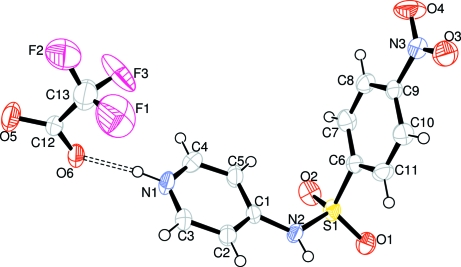
A view of the title compound with displacement ellipsoids drawn at the 50% probability level (arbitrary spheres for the H atoms). The dashed line indicates a hydrogen bond. Only the major component of the disordered CF3 group is shown.

## supplementary crystallographic information

### Comment

Organic pyridinium salts have been widely used in the construction of supramolecular architectures. As part of our ongoing studies of supramolecular chemistry involving the pyridinium rings (Li *et al.*, 2007), the structure of the title compound  was determined by X-ray diffraction.

In the cations of the title compound the short C—N distance is indicative of the slight conjugation of the sulphonamide N with the pyridinium ring. The benzene ring forms an angle of 87.3 (2)° with the pyridinium ring. The nitro group is essentially coplanar with the benzene ring

The crystal packing is stabilized by N—H···O and C—H···O hydrogen bonds.

### Experimental

4-Nitro-(*N*-pyridyl)benzenesulfonamide was prepared by the method of Yu & Li (2007). Crystals were obtained by   evaporation of a trifluoroacetic acid solution of the amide.

### Refinement

The N-bound H atoms were located in a difference map and their coordinates were refined. The C-bound H atoms were positioned geometrically (C—H = 0.93 Å) and refined as riding atoms. The constraint *U*_iso_(H) = 1.2 *U*_eq_(C and N) was applied. The N1—H1A distance was restrained at 0.90 (1) Å and C—F distances to 1.36 (1) Å. The instruction ISOR (tolerance 0.01) was applied to restrain the displacement ellipsoids of all F atoms to an isotropic bahaviour. The CF_3_ group  was disordered with the occupancy of 0.531 (12):0.469 (12).

### Crystal data

**Table:**

C_11_H_10_N_3_O_4_S^+^·C_2_F_3_O_2_^–^	*F*_000_ = 800
*M**_r_* = 393.30	*D*_x_ = 1.641 Mg m^−^^3^
Monoclinic, *P*2_1_/*c*	Mo *K*α radiation λ = 0.71073 Å
Hall symbol: -P 2ybc	Cell parameters from 1671 reflections
*a* = 5.662 (2) Å	θ = 2.5–24.6º
*b* = 17.497 (7) Å	µ = 0.28 mm^−^^1^
*c* = 16.173 (6) Å	*T* = 294 (2) K
β = 96.595 (7)º	Block, colourless
*V* = 1591.5 (11) Å^3^	0.10 × 0.04 × 0.04 mm
*Z* = 4	

### Data collection

**Table:**

Bruker SMART 1K CCD area-detector diffractometer	2763 independent reflections
Radiation source: fine-focus sealed tube	1596 reflections with *I* > 2σ(*I*)
Monochromator: graphite	*R*_int_ = 0.067
*T* = 294(2) K	θ_max_ = 25.0º
ω scans	θ_min_ = 1.7º
Absorption correction: multi-scan(SADABS; Sheldrick, 1996)	*h* = −6→6
*T*_min_ = 0.973, *T*_max_ = 0.989	*k* = −12→20
7696 measured reflections	*l* = −18→19

### Refinement

**Table:**

Refinement on *F*^2^	Secondary atom site location: difference Fourier map
Least-squares matrix: full	Hydrogen site location: inferred from neighbouring sites
*R*[*F*^2^ > 2σ(*F*^2^)] = 0.065	H atoms treated by a mixture of independent and constrained refinement
*wR*(*F*^2^) = 0.150	*w* = 1/[σ^2^(*F*_o_^2^) + (0.054*P*)^2^ + 1.2648*P*] where *P* = (*F*_o_^2^ + 2*F*_c_^2^)/3
*S* = 1.05	(Δ/σ)_max_ < 0.001
2763 reflections	Δρ_max_ = 0.40 e Å^−^^3^
269 parameters	Δρ_min_ = −0.33 e Å^−^^3^
43 restraints	Extinction correction: none
Primary atom site location: structure-invariant direct methods	

### Special details

**Table:**

Geometry. All e.s.d.'s (except the e.s.d. in the dihedral angle between two l.s. planes) are estimated using the full covariance matrix. The cell e.s.d.'s are taken into account individually in the estimation of e.s.d.'s in distances, angles and torsion angles; correlations between e.s.d.'s in cell parameters are only used when they are defined by crystal symmetry. An approximate (isotropic) treatment of cell e.s.d.'s is used for estimating e.s.d.'s involving l.s. planes.
Refinement. Refinement of *F*^2^ against ALL reflections. The weighted *R*-factor *wR* and goodness of fit *S* are based on *F*^2^, conventional *R*-factors *R* are based on *F*, with *F* set to zero for negative *F*^2^. The threshold expression of *F*^2^ > σ(*F*^2^) is used only for calculating *R*-factors(gt) *etc*. and is not relevant to the choice of reflections for refinement. *R*-factors based on *F*^2^ are statistically about twice as large as those based on *F*, and *R*- factors based on ALL data will be even larger.

### Fractional atomic coordinates and isotropic or equivalent isotropic displacement parameters (Å^2^)

**Table:**

	*x*	*y*	*z*	*U*_iso_*/*U*_eq_	Occ. (<1)
S1	1.1485 (2)	0.29446 (8)	0.83034 (7)	0.0340 (4)	
O1	1.1501 (6)	0.2461 (2)	0.90245 (19)	0.0469 (10)	
O2	1.3599 (5)	0.3060 (2)	0.7915 (2)	0.0466 (10)	
O3	0.6073 (7)	0.6017 (2)	0.9593 (2)	0.0536 (11)	
O4	0.8837 (7)	0.6574 (2)	0.9023 (3)	0.0648 (12)	
N1	0.7475 (8)	0.3207 (3)	0.5179 (2)	0.0418 (11)	
H1A	0.723 (8)	0.333 (3)	0.4637 (11)	0.050*	
N2	0.9372 (7)	0.2588 (3)	0.7627 (2)	0.0321 (10)	
H2A	0.853 (8)	0.229 (3)	0.790 (3)	0.039*	
N3	0.7809 (8)	0.6001 (3)	0.9208 (2)	0.0376 (11)	
C1	0.8800 (8)	0.2833 (3)	0.6816 (3)	0.0302 (12)	
C2	0.6688 (8)	0.2553 (3)	0.6379 (3)	0.0343 (12)	
H2	0.5692	0.2240	0.6648	0.041*	
C3	0.6093 (9)	0.2737 (3)	0.5565 (3)	0.0365 (13)	
H3	0.4718	0.2536	0.5276	0.044*	
C4	0.9464 (10)	0.3510 (3)	0.5585 (3)	0.0440 (14)	
H4	1.0385	0.3839	0.5304	0.053*	
C5	1.0155 (9)	0.3343 (3)	0.6397 (3)	0.0388 (13)	
H5	1.1515	0.3566	0.6673	0.047*	
C6	1.0399 (8)	0.3856 (3)	0.8545 (3)	0.0290 (11)	
C7	1.1511 (9)	0.4521 (3)	0.8325 (3)	0.0392 (13)	
H7	1.2837	0.4490	0.8036	0.047*	
C8	1.0671 (9)	0.5226 (3)	0.8528 (3)	0.0392 (13)	
H8	1.1409	0.5673	0.8382	0.047*	
C9	0.8688 (8)	0.5251 (3)	0.8959 (3)	0.0297 (11)	
C10	0.7523 (8)	0.4602 (3)	0.9186 (3)	0.0358 (12)	
H10	0.6186	0.4639	0.9468	0.043*	
C11	0.8397 (8)	0.3893 (3)	0.8981 (3)	0.0366 (13)	
H11	0.7663	0.3447	0.9132	0.044*	
C12	0.5532 (9)	0.3961 (3)	0.3109 (3)	0.0337 (12)	
O5	0.5106 (7)	0.4020 (2)	0.2352 (2)	0.0588 (12)	
O6	0.6749 (6)	0.3465 (2)	0.35187 (18)	0.0418 (9)	
C13	0.4492 (13)	0.4576 (4)	0.3613 (4)	0.075 (2)	
F1	0.320 (2)	0.4376 (10)	0.4198 (8)	0.117 (6)	0.531 (12)
F2	0.367 (2)	0.5185 (5)	0.3176 (6)	0.101 (4)	0.531 (12)
F3	0.6434 (17)	0.4927 (6)	0.4089 (7)	0.115 (5)	0.531 (12)
F1'	0.2000 (16)	0.4600 (8)	0.3372 (7)	0.110 (5)	0.469 (12)
F2'	0.512 (3)	0.5285 (6)	0.3520 (10)	0.116 (5)	0.469 (12)
F3'	0.430 (2)	0.4372 (9)	0.4392 (5)	0.080 (4)	0.469 (12)

### Atomic displacement parameters (Å^2^)

**Table:**

	*U*^11^	*U*^22^	*U*^33^	*U*^12^	*U*^13^	*U*^23^
S1	0.0332 (7)	0.0405 (8)	0.0257 (6)	0.0051 (7)	−0.0073 (5)	0.0002 (6)
O1	0.059 (2)	0.046 (2)	0.0306 (19)	0.0025 (19)	−0.0152 (17)	0.0062 (17)
O2	0.0287 (18)	0.058 (3)	0.052 (2)	0.0050 (18)	0.0021 (16)	−0.010 (2)
O3	0.060 (2)	0.050 (3)	0.055 (2)	0.007 (2)	0.023 (2)	0.002 (2)
O4	0.073 (3)	0.033 (2)	0.091 (3)	−0.016 (2)	0.020 (2)	0.006 (2)
N1	0.058 (3)	0.047 (3)	0.020 (2)	0.004 (2)	0.001 (2)	0.004 (2)
N2	0.037 (2)	0.037 (3)	0.022 (2)	−0.006 (2)	0.0007 (17)	0.0050 (18)
N3	0.044 (3)	0.038 (3)	0.030 (2)	0.000 (2)	0.004 (2)	0.002 (2)
C1	0.040 (3)	0.029 (3)	0.021 (2)	0.002 (2)	0.003 (2)	−0.004 (2)
C2	0.037 (3)	0.038 (3)	0.027 (2)	−0.004 (3)	0.004 (2)	−0.002 (2)
C3	0.044 (3)	0.041 (3)	0.024 (2)	−0.003 (3)	−0.001 (2)	−0.001 (2)
C4	0.055 (3)	0.044 (4)	0.035 (3)	−0.006 (3)	0.012 (3)	0.007 (3)
C5	0.044 (3)	0.047 (4)	0.025 (3)	−0.005 (3)	0.000 (2)	−0.002 (2)
C6	0.028 (3)	0.033 (3)	0.024 (2)	−0.001 (2)	−0.005 (2)	0.001 (2)
C7	0.033 (3)	0.048 (4)	0.037 (3)	−0.004 (3)	0.009 (2)	−0.005 (3)
C8	0.044 (3)	0.042 (4)	0.033 (3)	−0.015 (3)	0.011 (2)	0.002 (3)
C9	0.033 (3)	0.033 (3)	0.023 (2)	−0.002 (2)	−0.002 (2)	0.000 (2)
C10	0.028 (3)	0.042 (4)	0.037 (3)	−0.005 (3)	0.007 (2)	−0.001 (3)
C11	0.039 (3)	0.033 (3)	0.038 (3)	−0.010 (3)	0.003 (2)	0.005 (3)
C12	0.044 (3)	0.034 (3)	0.024 (3)	−0.004 (3)	0.006 (2)	0.000 (2)
O5	0.083 (3)	0.067 (3)	0.026 (2)	0.015 (2)	0.0016 (19)	0.0039 (19)
O6	0.056 (2)	0.045 (2)	0.0227 (18)	0.0032 (19)	−0.0004 (16)	−0.0023 (17)
C13	0.107 (6)	0.071 (6)	0.045 (4)	0.018 (5)	0.006 (4)	0.001 (4)
F1	0.110 (9)	0.136 (9)	0.117 (9)	0.002 (7)	0.059 (7)	−0.026 (7)
F2	0.142 (8)	0.060 (6)	0.098 (6)	0.050 (6)	0.005 (6)	−0.013 (5)
F3	0.138 (8)	0.092 (7)	0.114 (7)	−0.003 (6)	0.008 (6)	−0.076 (6)
F1'	0.090 (7)	0.138 (9)	0.105 (7)	0.044 (6)	0.022 (5)	−0.024 (6)
F2'	0.131 (9)	0.079 (8)	0.142 (9)	−0.010 (7)	0.031 (7)	−0.018 (7)
F3'	0.093 (8)	0.097 (7)	0.053 (5)	0.027 (7)	0.023 (5)	−0.026 (5)

### Geometric parameters (Å, °)

**Table:**

S1—O2	1.428 (3)	C6—C7	1.388 (7)
S1—O1	1.440 (3)	C6—C11	1.404 (6)
S1—N2	1.648 (4)	C7—C8	1.376 (7)
S1—C6	1.769 (5)	C7—H7	0.9300
O3—N3	1.222 (5)	C8—C9	1.388 (6)
O4—N3	1.213 (5)	C8—H8	0.9300
N1—C3	1.338 (6)	C9—C10	1.384 (6)
N1—C4	1.345 (6)	C10—C11	1.389 (7)
N1—H1A	0.899 (11)	C10—H10	0.9300
N2—C1	1.382 (5)	C11—H11	0.9300
N2—H2A	0.86 (5)	C12—O5	1.225 (5)
N3—C9	1.476 (6)	C12—O6	1.250 (5)
C1—C5	1.401 (7)	C12—C13	1.511 (8)
C1—C2	1.405 (6)	C13—F2'	1.303 (9)
C2—C3	1.359 (6)	C13—F1	1.309 (9)
C2—H2	0.9300	C13—F3'	1.326 (9)
C3—H3	0.9300	C13—F2	1.333 (8)
C4—C5	1.359 (7)	C13—F3	1.408 (8)
C4—H4	0.9300	C13—F1'	1.420 (8)
C5—H5	0.9300		
			
O2—S1—O1	120.8 (2)	C7—C8—C9	118.0 (5)
O2—S1—N2	110.0 (2)	C7—C8—H8	121.0
O1—S1—N2	104.6 (2)	C9—C8—H8	121.0
O2—S1—C6	107.4 (2)	C10—C9—C8	123.0 (5)
O1—S1—C6	108.7 (2)	C10—C9—N3	118.1 (4)
N2—S1—C6	104.1 (2)	C8—C9—N3	118.8 (5)
C3—N1—C4	121.0 (4)	C9—C10—C11	118.4 (4)
C3—N1—H1A	125 (3)	C9—C10—H10	120.8
C4—N1—H1A	114 (3)	C11—C10—H10	120.8
C1—N2—S1	125.8 (4)	C10—C11—C6	119.4 (5)
C1—N2—H2A	126 (3)	C10—C11—H11	120.3
S1—N2—H2A	107 (3)	C6—C11—H11	120.3
O4—N3—O3	122.9 (5)	O5—C12—O6	128.5 (5)
O4—N3—C9	118.7 (4)	O5—C12—C13	115.8 (5)
O3—N3—C9	118.4 (4)	O6—C12—C13	115.7 (4)
N2—C1—C5	125.1 (4)	F2'—C13—F1	121.4 (11)
N2—C1—C2	117.6 (4)	F2'—C13—F3'	114.8 (11)
C5—C1—C2	117.4 (4)	F1—C13—F3'	29.3 (8)
C3—C2—C1	120.5 (5)	F2'—C13—F2	42.4 (7)
C3—C2—H2	119.8	F1—C13—F2	114.0 (10)
C1—C2—H2	119.8	F3'—C13—F2	131.1 (9)
N1—C3—C2	120.3 (5)	F2'—C13—F3	56.1 (8)
N1—C3—H3	119.9	F1—C13—F3	100.8 (9)
C2—C3—H3	119.9	F3'—C13—F3	75.0 (8)
N1—C4—C5	121.3 (5)	F2—C13—F3	98.4 (9)
N1—C4—H4	119.4	F2'—C13—F1'	102.5 (10)
C5—C4—H4	119.4	F1—C13—F1'	65.5 (8)
C4—C5—C1	119.4 (5)	F3'—C13—F1'	94.7 (9)
C4—C5—H5	120.3	F2—C13—F1'	63.6 (7)
C1—C5—H5	120.3	F3—C13—F1'	145.7 (8)
C7—C6—C11	120.5 (5)	F2'—C13—C12	119.2 (8)
C7—C6—S1	121.3 (4)	F1—C13—C12	119.0 (9)
C11—C6—S1	118.2 (4)	F3'—C13—C12	113.6 (8)
C8—C7—C6	120.7 (5)	F2—C13—C12	114.7 (6)
C8—C7—H7	119.7	F3—C13—C12	106.1 (6)
C6—C7—H7	119.7	F1'—C13—C12	108.0 (6)
			
O2—S1—N2—C1	−44.4 (5)	C7—C8—C9—N3	178.0 (4)
O1—S1—N2—C1	−175.5 (4)	O4—N3—C9—C10	178.9 (4)
C6—S1—N2—C1	70.4 (4)	O3—N3—C9—C10	−1.0 (6)
S1—N2—C1—C5	10.6 (7)	O4—N3—C9—C8	0.5 (6)
S1—N2—C1—C2	−169.1 (4)	O3—N3—C9—C8	−179.4 (4)
N2—C1—C2—C3	−176.2 (5)	C8—C9—C10—C11	0.8 (7)
C5—C1—C2—C3	4.1 (7)	N3—C9—C10—C11	−177.6 (4)
C4—N1—C3—C2	−0.4 (8)	C9—C10—C11—C6	−0.8 (7)
C1—C2—C3—N1	−2.1 (8)	C7—C6—C11—C10	0.5 (7)
C3—N1—C4—C5	0.7 (8)	S1—C6—C11—C10	179.4 (4)
N1—C4—C5—C1	1.5 (8)	O5—C12—C13—F2'	−60.9 (12)
N2—C1—C5—C4	176.6 (5)	O6—C12—C13—F2'	118.0 (11)
C2—C1—C5—C4	−3.8 (7)	O5—C12—C13—F1	126.9 (10)
O2—S1—C6—C7	2.8 (4)	O6—C12—C13—F1	−54.3 (12)
O1—S1—C6—C7	135.0 (4)	O5—C12—C13—F3'	159.0 (9)
N2—S1—C6—C7	−113.9 (4)	O6—C12—C13—F3'	−22.1 (11)
O2—S1—C6—C11	−176.1 (3)	O5—C12—C13—F2	−13.2 (11)
O1—S1—C6—C11	−43.8 (4)	O6—C12—C13—F2	165.7 (9)
N2—S1—C6—C11	67.2 (4)	O5—C12—C13—F3	−120.7 (7)
C11—C6—C7—C8	−0.1 (7)	O6—C12—C13—F3	58.2 (8)
S1—C6—C7—C8	−178.9 (4)	O5—C12—C13—F1'	55.4 (9)
C6—C7—C8—C9	0.0 (7)	O6—C12—C13—F1'	−125.8 (8)
C7—C8—C9—C10	−0.4 (7)		

### Hydrogen-bond geometry (Å, °)

**Table:**

*D*—H···*A*	*D*—H	H···*A*	*D*···*A*	*D*—H···*A*
N1—H1A···O6	0.899 (11)	1.812 (14)	2.706 (5)	173 (5)
N2—H2A···O6^i^	0.86 (5)	2.00 (5)	2.858 (5)	176 (5)
C2—H2···O5^i^	0.93	2.52	3.343 (6)	148
C3—H3···O1^ii^	0.93	2.56	3.406 (6)	151
C8—H8···O5^iii^	0.93	2.48	3.204 (6)	135
C10—H10···O3^iv^	0.93	2.39	3.184 (6)	143

Symmetry codes: (i) *x*, −*y*+1/2, *z*+1/2; (ii) *x*−1, −*y*+1/2, *z*−1/2; (iii) −*x*+2, −*y*+1, −*z*+1; (iv) −*x*+1, −*y*+1, −*z*+2.

## References

[bb1] Bruker (1997). *SMART* and *SAINT* Bruker AXS Inc., Madison, Wisconsin, USA.

[bb2] Li, J. S., Chen, L. G., Zhang, Y. Y., Xu, Y. J., Deng, Y. & Huang, P. M. (2007). *J. Chem. Res.* pp. 350–352.

[bb3] Sheldrick, G. M. (1996). *SADABS* University of Göttingen, Germany.

[bb4] Sheldrick, G. M. (2008). *Acta Cryst.* A**64**, 112–122. 10.1107/S0108767307043930 18156677

[bb5] Yu, H.-J. & Li, J.-S. (2007). *Acta Cryst.* E**63**, o3399.

[bb6] Zhou, B., Zheng, P.-W., Liu, K.-Y. & Cheng, D. (2008). *Acta Cryst.* E**64**, o254. 10.1107/S160053680706504X PMC2915311 21200819

